# *Francisella tularensis* disrupts TLR2-MYD88-p38 signaling early during infection to delay apoptosis of macrophages and promote virulence in the host

**DOI:** 10.1128/mbio.01136-23

**Published:** 2023-07-05

**Authors:** P. Todd Benziger, Erik J. Kopping, Patrick A. McLaughlin, David G. Thanassi

**Affiliations:** 1 Department of Microbiology and Immunology, Renaissance School of Medicine, Stony Brook University, Stony Brook, New York, USA; 2 Center for Infectious Diseases, Stony Brook University, Stony Brook, New York, USA; Universite de Geneve, Genève, Switzerland

**Keywords:** bacterial pathogenesis, *Francisella tularensis*, macrophages, apoptosis

## Abstract

*Francisella tularensis* is a zoonotic pathogen and the causative agent of tularemia. *F. tularensis* replicates to high levels within the cytosol of macrophages and other host cells while subverting the host response to infection. Critical to the success of *F. tularensis* is its ability to delay macrophage apoptosis to maintain its intracellular replicative niche. However, the host-signaling pathway(s) modulated by *F. tularensis* to delay apoptosis are poorly characterized. The outer membrane channel protein TolC is required for *F. tularensis* virulence and its ability to suppress apoptosis and cytokine expression during infection of macrophages. We took advantage of the *F. tularensis* ∆*tolC* mutant phenotype to identify host pathways that are important for activating macrophage apoptosis and that are disrupted by the bacteria. Comparison of macrophages infected with wild-type or ∆*tolC F. tularensis* revealed that the bacteria interfere with TLR2-MYD88-p38 signaling at early times post infection to delay apoptosis, dampen innate host responses, and preserve the intracellular replicative niche. Experiments using the mouse pneumonic tularemia model confirmed the *in vivo* relevance of these findings, revealing contributions of TLR2 and MYD88 signaling to the protective host response to *F. tularensis*, which is modulated by the bacteria to promote virulence*.*

**IMPORTANCE:**

*Francisella tularensis* is a Gram-negative intracellular bacterial pathogen and the causative agent of the zoonotic disease tularemia. *F. tularensis*, like other intracellular pathogens, modulates host-programmed cell death pathways to ensure its replication and survival. We previously identified the outer membrane channel protein TolC as required for the ability of *F. tularensis* to delay host cell death. However, the mechanism by which *F. tularensis* delays cell death pathways during intracellular replication is unclear despite being critical to pathogenesis. In the present study, we address this gap in knowledge by taking advantage of ∆*tolC* mutants of *F. tularensis* to uncover signaling pathways governing host apoptotic responses to *F. tularensis* and which are modulated by the bacteria during infection to promote virulence. These findings reveal mechanisms by which intracellular pathogens subvert host responses and enhance our understanding of the pathogenesis of tularemia.

## INTRODUCTION

*Francisella tularensis* is a Gram-negative, facultative intracellular bacterium and the causative agent of the zoonotic disease tularemia. Direct inhalation of the bacteria leads to highly lethal pneumonia, with mortality rates as high as 60% if left untreated (1). Due to its high lethality, ease of infection, and potential for misuse as a biological agent, *F. tularensis* is classified as a Tier 1 select agent by the Centers for Disease Control and Prevention. Within North America, there are two clinically important *F. tularensis* subspecies: subsp. *tularensis* (Type A) and subsp. *holarctica* (Type B). Subsp. *tularensis* is more virulent in humans compared to subsp. *holarctica* (1). An attenuated *F. tularensis* live vaccine strain (LVS), derived from subsp. *holarctica*, causes lethal infection in mice that closely mimics the pathogenesis of human virulent strains, making it a valuable experimental tool (1). The LVS is not licensed for use as a vaccine in the United States because its mechanism of attenuation is not completely understood and it does not fully protect against infection with subsp. *tularensis* strains. Therefore, there is a need to better understand *F. tularensis* virulence mechanisms and host responses to infection, which will facilitate the development of improved vaccines and therapeutic approaches.

*F. tularensis* primarily replicates within macrophages early during infection (2). Upon phagocytosis, the bacteria escape the phagosome as early as 30 min post infection (p.i.) and then begin replicating in the host cell cytosol. After several rounds of intracellular replication, host cell death is induced, contributing to bacterial dissemination and further infection (3 - 6). Critical to the success of *F. tularensis* is its ability to remain immunologically silent during early replication and spread within the host. In mouse pneumonic tularemia models, strong inflammatory responses are not observed until after the first 2–3 d p.i (7 - 9). Similar observations have been made in the human disease (10 - 12). To evade detection by the host, *F. tularensis* actively interferes with host innate immune responses by dampening pro-inflammatory cytokine expression and delaying programmed cell death pathways (5, 13 - 21). During infection of macrophages, *F. tularensis* delays host cell death until late time points (~24 h p.i.), at a stage where the bacteria have replicated to high levels; the induction of apoptosis then allows bacterial spread under non-inflammatory conditions. Despite being critical to virulence, the mechanism by which *Francisella* delays apoptosis during infection is poorly understood (18, 19, 21 - 24).

Several host receptors and signaling pathways have been identified as important for control of *F. tularensis* infection. The LPS of *F. tularensis* does not stimulate Toll-like receptor 4 (TLR4), and thus, TLR4 is dispensable for host protection (25). Instead, TLR2 is the best-characterized pattern recognition receptor responsible for pro-inflammatory responses to *F. tularensis* (26 - 30). Activation of TLR2 leads to recruitment of the signaling adapter molecule Myeloid Differentiation Factor 88 (MYD88). The binding of MYD88 then recruits downstream effectors and kinases into a complex called the Myddosome, to initiate and tune the immune response to the impending danger (31). Key intracellular signaling networks activated downstream of the Myddosome include the Mitogen-Activated Protein Kinase (MAPK) and NF-κB pathways. During pathogen recognition, the MAPK families p38, SAPK/JNK, and ERK1/2 regulate both pro-inflammatory cytokine expression and programmed cell death pathways. Activation of p38 and SAPK/JNK generally promotes apoptosis, whereas ERK1/2 and NF-κB signaling is associated with cell survival (32). The NF-κB pathway and all three MAPKs (P38, SAPK/JNK, ERK1/2) mediate host inflammatory responses to *F. tularensis* in a TLR2-dependent manner (33 - 37). Additionally, MYD88 functions in protection against *F. tularensis* independent of TLR2 by regulating expression of interferon-γ (IFN-γ) downstream of the interleukin 1 receptor (IL-1R) (38 - 40).

We previously identified the *F. tularensis* TolC protein as a virulence factor of both the LVS and human virulent Schu S4 strain that is critical for the ability to delay apoptosis and dampen pro-inflammatory cytokine expression during infection (21, 23, 41). TolC belongs to a class of Gram-negative bacterial outer membrane channel proteins involved in the type I protein secretion pathway and efflux of small molecules (42). *F. tularensis* TolC deletion mutants elicit increased release of pro-inflammatory cytokines and increased cytotoxicity during infection of macrophages, corresponding to premature activation of the apoptotic pathway and loss of the intracellular replicative niche (21, 23, 41, 43). Both the LVS and Schu S4 ∆*tolC* mutants are highly attenuated in mouse tularemia infection models (21, 23, 41). While the ∆*tolC* mutants are competent for phagosomal escape, intracellular replication, and dissemination to distal organs during *in vivo* infection, compared to the wild-type (WT) bacteria, the mutants activate increased caspase-3 cleavage, replicate to lower numbers, and are eventually cleared by the host (21, 23, 41, 43).

In the current study, we utilized ∆*tolC* mutants in both the LVS and Schu S4 strain to uncover signaling pathways by which the host recognizes *F. tularensis* to control infection. Comparison of primary murine macrophages infected with WT or ∆*tolC* LVS revealed that *F. tularensis* disrupts MAPK signaling at early times p.i. to reduce cytotoxicity and preserve its intracellular replicative niche. Specifically, we present evidence that the detection of *F. tularensis* and subsequent phosphorylation of the p38 MAPK is mediated by TLR2-MYD88 signaling, which activates the host apoptotic response. Furthermore, we found that the human virulent Schu S4 strain similarly modulates TLR2- and MAPK-dependent signaling early during infection to dampen macrophage cell death and pro-inflammatory cytokine responses. Finally, experiments using the mouse pneumonic tularemia model revealed that both TLR2 and MYD88 signaling contribute to the early host response to LVS infection *in vivo* and to the protection against lethal infection. Notably, the LVS ∆*tolC* mutant regained full virulence in MYD88^−/−^ mice, identifying a critical protective role for MYD88-dependent signaling in the pathogenesis of tularemia.

## RESULTS

### *F. tularensis* dampens MAPK signaling in macrophages to reduce cytotoxicity during intracellular infection

**Fig 1:**
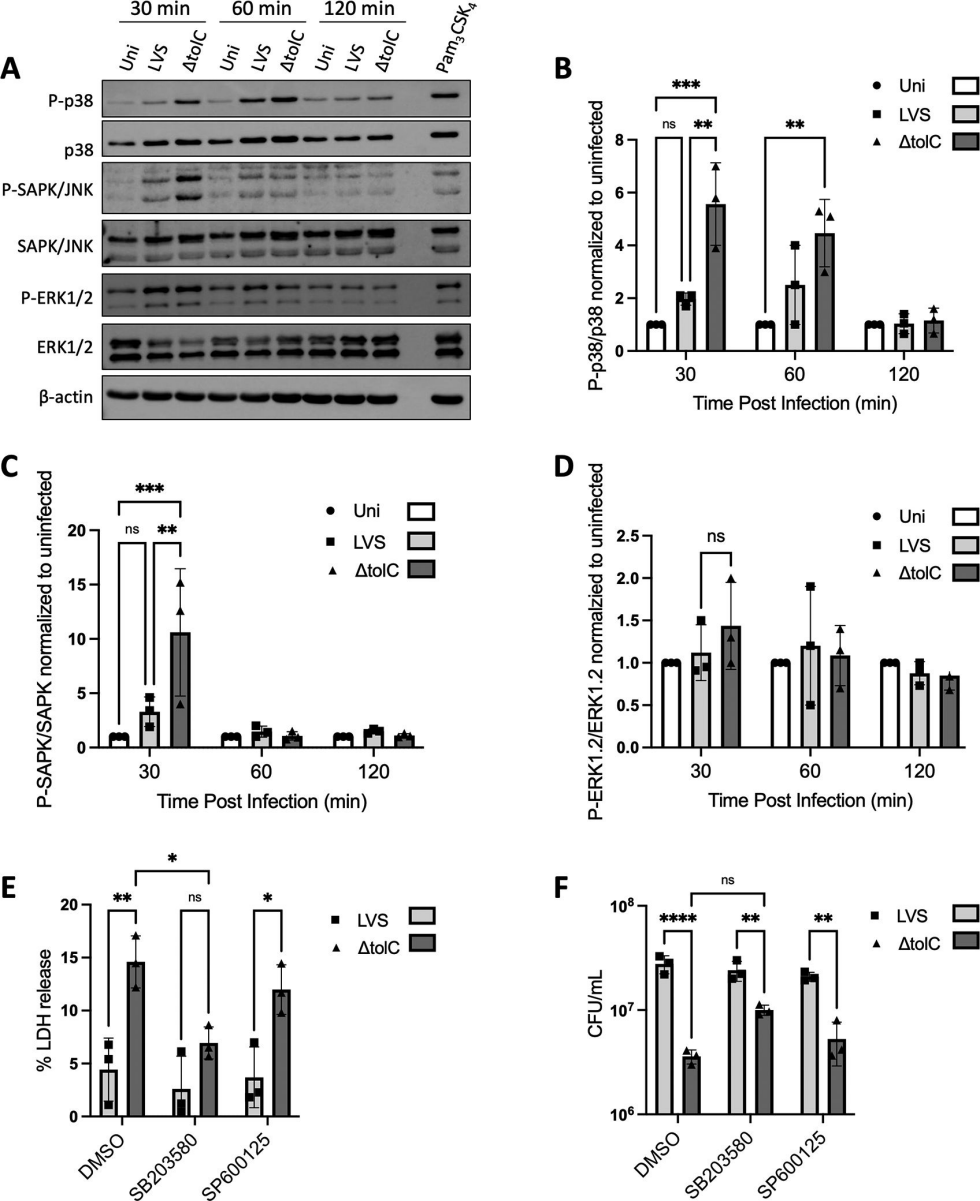
MAPK activation during LVS infection and role in cytotoxicity and intracellular replication. (**A**) BMMs were infected at an MOI of 50 with the WT or Δ*tolC* LVS or left uninfected. At the indicated times, macrophage lysates were collected and assayed for phosphorylation of p38, SAPK/JNK, and ERK1/2 by SDS-PAGE and immunoblotting. As a positive control, BMMs were treated with 25 ng/µL of Pam_3_CSK_4_ for 1 h. A representative blot is shown. (B–D) Ratio of phosphorylated to unphosphorylated protein for p38 (**B**), SAPK/JNK (**C**), and ERK1/2 (**D**), relative to the uninfected control, determined from densitometric analysis of blots from three independent experiments. (**E and F**) BMMs were pretreated for 30 min with 10 µM of the p38 inhibitor SB203580, 15 µM of the SAPK/JNK inhibitor SP62001, or DMSO as a vehicle control, and then infected at an MOI of 50 with the WT or Δ*tolC* LVS. At 24 h p.i., supernatant fractions were collected and assayed for levels of LDH release (**E**) or cell lysates were plated for colony-forming units (CFUs) (**F**). Data in (B–F) represent means ± SEM of three independent experiments. *P < 0.05; **P < 0.01; ***P < 0.001; ****P < 0.0001; ns, not significant; calculated by two-way ANOVA test with Tukey’s multiple-comparison posttest.

*F. tularensis* delays the apoptotic response of infected macrophages to preserve its intracellular replicative niche (5, 19, 21 - 24). We previously demonstrated that this delay in apoptosis is dependent on the TolC protein (21, 23). We, therefore, took advantage of the LVS ∆*tolC* mutant to probe host cell signaling pathways modulated during infection. Given the role of MAPKs in the host response to *F. tularensis* (33 - 37), we examined if infection of murine bone marrow-derived macrophages (BMMs) with the LVS Δ*tolC* mutant leads to increased p38, SAPK/JNK, or ERK1/2 activation in comparison to infection with the WT LVS. BMMs were infected with either the WT or Δ*tolC* LVS, or left uninfected, and MAPK activation (i.e., phosphorylation) was measured via immunoblot analysis of cell lysates. As shown in Fig. 1A through D, infection with the WT LVS did not cause significant increases in phosphorylation of the MAPKs compared to the uninfected controls. In contrast, infection with the ∆*tolC* LVS triggered increased levels of phosphorylated p38 and SAPK/JNK at 30 min p.i. compared to both the WT LVS-infected macrophages and uninfected controls (Fig. 1A through D). These differences were no longer observed by 60 and 120 min p.i. and were also not observed at later time points (12 h p.i.; data not shown). In contrast to p38 and SAPK/JNK, we did not observe differences in levels of phospho-ERK1/2 between the infected BMMs and uninfected controls (Fig. 1A and D). These results demonstrate that both the p38 and SAPK/JNK MAPK pathways are dampened by *F. tularensis*, via a TolC-dependent mechanism, during the early macrophage response to infection.

Both the p38 and SAPK/JNK MAPK pathways are important for regulating apoptosis (32, 44, 45). To determine if activation of p38 and SAPK/JNK during *F. tularensis* infection impacts the macrophage apoptotic response, BMMs were treated with the inhibitors SB203580 or SP600125, which target p38 or SAPK/JNK, respectively (36, 46 - 50). BMMs were treated with inhibitor, or DMSO as a control, and at 24 h p.i., release of lactate dehydrogenase (LDH) was measured as a marker for host cell death and CFUs were determined to measure intracellular bacterial replication. In the control BMMs, infection with the Δ*tolC* LVS resulted in an approximately threefold increase in host cell death and~1 log decrease in bacterial replication compared to infection with the WT LVS (Fig. 1E and F). Thus, as previously observed (21, 41), the *F. tularensis* ∆*tolC* mutant is defective in dampening host cell death and, as a result, exhibits decreased intracellular replication. Treatment of BMMs with the SAPK/JNK inhibitor SP600125 did not alter these ∆*tolC* mutant phenotypes (Fig. 1E and F). In contrast, for BMMs treated with the p38 inhibitor SB230580, infection with the ∆*tolC* mutant resulted in decreased host cell death, and the intracellular replication defect of the mutant was diminished (Fig. 1E and F). Together, these results implicate p38 in the macrophage apoptotic response to *F. tularensis* infection and suggest that the bacteria dampen this response early during infection to preserve the intracellular niche.

### P38 phosphorylation and the downstream apoptotic response occur via a TLR2-MYD88 signaling pathway

**Fig 2:**
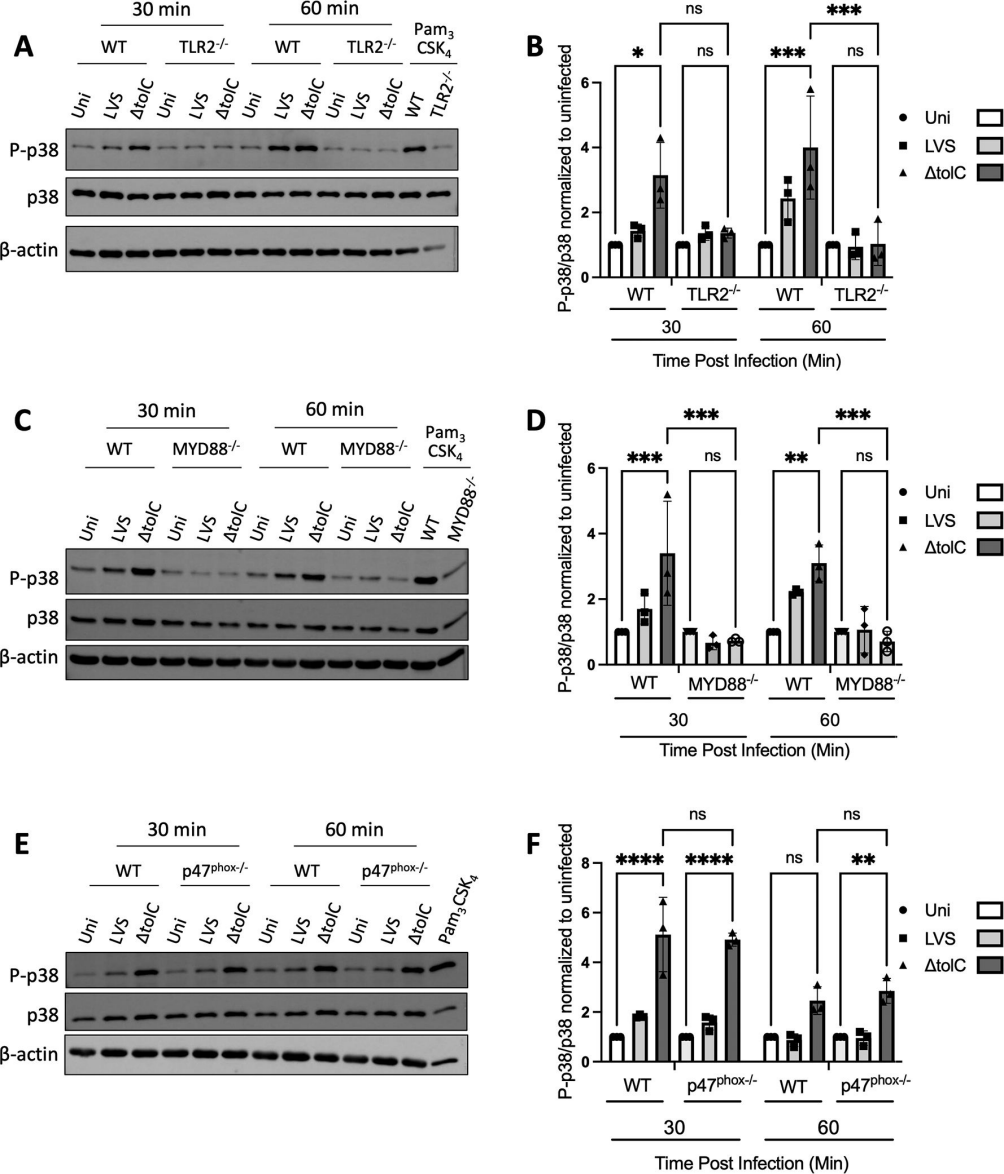
TLR2 and MYD88 are required for p38 phosphorylation during LVS infection. BMMs isolated from WT and TLR2^−/−^ (**A**), MYD88^−/−^ (**C**), or p47^phox−/−^ (**E**) mice were infected at an MOI of 50 with the WT or Δ*tolC* LVS or left uninfected. At the indicated times, macrophage lysates were collected and assayed for phosphorylation of p38 by SDS-PAGE and immunoblotting. As a positive control, BMMs were treated with 25 ng/µL of Pam_3_CSK_4_ for 1 h. Representative blots are shown. (**B, D, F**) Ratio of phosphorylated to unphosphorylated p38, relative to the uninfected control, determined from densitometric analysis of corresponding blots from three independent experiments. Data represent means ± SEM. *P < 0.05; **P < 0.01; ***P < 0.001; *P* < 0.0001; ns, not significant; calculated by two-way ANOVA test with Tukey’s multiple-comparison posttest.

Activation of p38 in response to microbial infection occurs downstream of TLR-signaling pathways (45). To determine if TLR2 is required for activation of p38 during *F. tularensis* infection, we compared BMMs isolated from WT or TLR2^−/−^ mice. BMMs were infected with the WT or ∆*tolC* LVS, or left uninfected, and then probed for phosho-p38. In agreement with the preceding results, increased p38 phosphorylation was detected for the WT BMMs infected with the Δ*tolC* mutant at early times p.i. (Fig. 2A and B). However, no increased p38 phosphorylation was observed for the TLR2^−/−^ BMMs (Fig. 2A and B). To test if MYD88 is required together with TLR2 for MAPK activation in response to *F. tularensis* infection, we examined p38 phosphorylation in BMMs isolated from WT or MYD88^−/−^ mice. Similar to the TLR2^−/−^ macrophages, increased p38 phosphorylation was no longer observed for the MYD88^−/−^ BMMs infected with the Δ*tolC* LVS (Fig. 2C and D). *F. tularensis* can also activate p38 through redox signaling via reactive oxygen species (ROS) produced by the NADPH oxidase (NOX2) (13). In macrophages, the production of ROS by NOX2 occurs downstream of TLR activation and requires the p47^phox^ subunit (51, 52). However, in contrast to infection of the TLR2^−/−^ and MYD88^−/−^ macrophages, infection of p47^phox−/−^ BMMs with the ∆*tolC* LVS still resulted in increased p38 phosphorylation at 30 and 60 min p.i. (Fig. 2E and F). Together, these results confirm that macrophages sense *F. tularensis* infection via TLR2 and demonstrate that the bacteria interfere with the TLR2-MYD88 pathway, independent of the host NADPH oxidase, to prevent p38 signaling.

**Fig 3:**
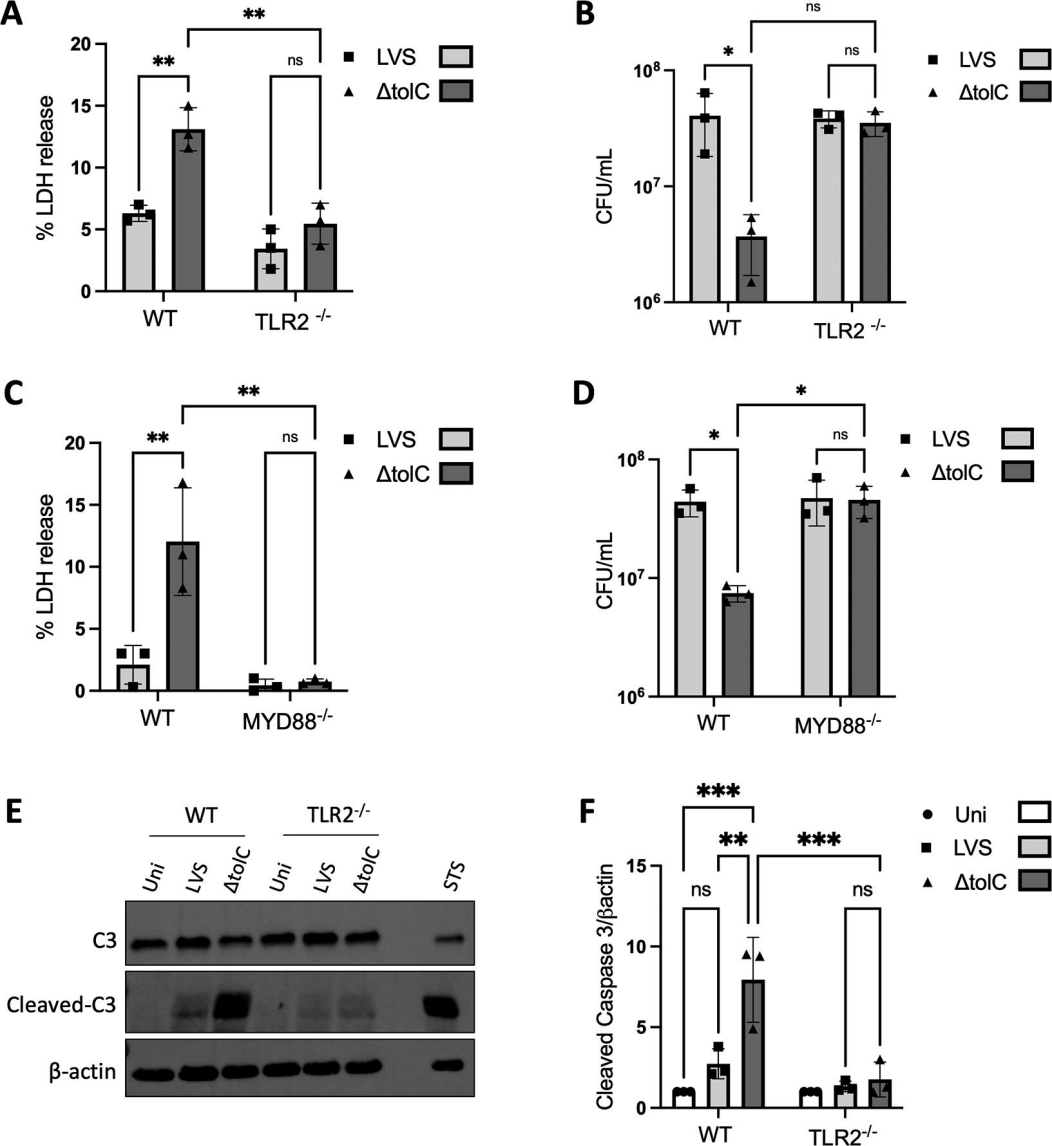
Requirement for TLR2 and MYD88 in cytotoxicity, intracellular replication, and apoptosis during LVS infection. BMMs isolated from WT and TLR2^−/−^ (**A, B, E, F**) or MYD88^−/−^ (**C and D**) mice were infected at an MOI of 50 with the WT or ∆*tolC* LVS or left uninfected. At 24 h p.i., supernatant fractions were collected and assayed for levels of LDH release (**A and C**), or cell lysates were plated for CFUs (**B and D**). (**E**) At 18 h p.i., macrophage lysates were collected and assayed for cleavage of caspase-3, as a marker for apoptosis, by SDS-PAGE and immunoblotting. As a positive control, BMMs were treated with staurosporine for 5 h. A representative blot is shown. (**F**) Ratio of cleaved capsase-3 to β-actin, relative to the uninfected control, determined from densitometric analysis of blots from three independent experiments. Data in (A–D and **F**) represent means ± SEM of three independent experiments. *P < 0.05; **P < 0.01; ***P < 0.001; ns, not significant; calculated by two-way ANOVA test with Tukey’s multiple-comparison posttest.

We next examined the connection of TLR2-MYD88 signaling to the downstream macrophage cell death response to *F. tularensis* infection. BMMs isolated from WT, TLR2^−/−^ or MYD88^−/−^ mice were infected with WT or Δ*tolC* LVS, and host cell death was measured by LDH release at 24 h p.i. Whereas infection of WT BMMs with the ∆*tolC* mutant led to an approximately twofold increase in cell death compared to infection with the WT LVS, this was no longer observed for the TLR2^−/−^ and MYD88^−/−^ BMMs (Fig. 3A and C). In agreement with these results, intracellular replication of the ∆*tolC* mutant was similar to the WT LVS in both the TLR2^−/−^ and MYD88^−/−^ BMMs (Fig. 3B and D). Thus, the intracellular replicative niche for the ∆*tolC* mutant is preserved in the absence of host cell signaling through TLR2 and MYD88. No changes in the host cell death or intracellular replication phenotypes of the LVS ∆*tolC* mutant were observed during infection of p47^phox−/−^ BMMs (Fig. S1). The LVS ∆*tolC* mutant triggers activation of the apoptotic pathway as early as 6 h p.i., whereas WT bacteria actively suppress and delay this apoptotic response (21). To determine if TLR2 signaling contributes to the downstream apoptotic response during *F. tularensis* infection, we compared levels of cleaved caspase-3 in WT or TLR2^−/−^ BMMs infected with the WT or Δ*tolC* LVS. In WT BMMs, increased caspase-3 cleavage was detected at 18 h p.i. in response to the Δ*tolC* mutant when compared to infection with the WT bacteria or uninfected control (Fig. 3E and F). In contrast, no difference in caspase-3 cleavage was observed for infection of the TLR2^−/−^ macrophages (Fig. 3E and F). Thus, *F. tularensis* delays macrophage apoptosis by dampening TLR2 signaling via a TolC-dependent mechanism.

### *F. tularensis* Schu S4 modulates MAPK signaling and TLR2-dependent innate immune responses during macrophage infection

**Fig 4:**
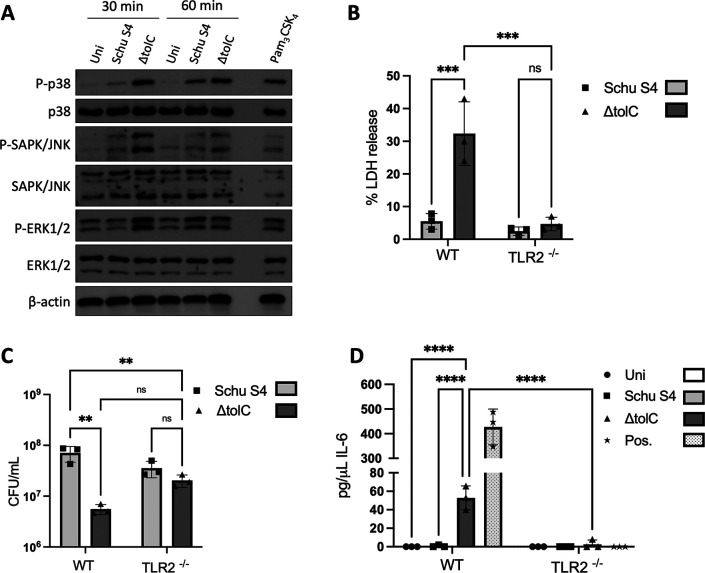
MAPK activation during Schu S4 infection and role of TLR2 in cytotoxicity, intracellular replication, and pro-inflammatory cytokine release. (**A**) BMMs were infected at an MOI of 500 with WT or Δt*olC* SCHU S4 or left uninfected. At the indicated time points, macrophage lysates were collected and assayed for phosphorylation of p38, SAPK/JNK, and ERK1/2 by SDS-PAGE and immunoblotting. As a positive control, BMMs were treated with 25 ng/µL of Pam_3_CSK_4_ for 1 h. A representative blot is shown; an additional blot is shown in Fig. S2. (B–D) BMMs isolated from WT and TLR2^−/−^ mice were infected at an MOI of 500 with either the WT or ∆*tolC* SCHU S4 or left uninfected. At 22 h p.i., supernatant fractions were collected and assayed for levels of LDH release (**B**) or cell lysates were plated for CFUs (**C**). (**D**) At 22 h p.i., supernatant fractions were collected and assayed for secretion of IL-6 by ELISA. As a positive control, BMMs were treated with 25 ng/µL of Pam_3_CSK_4_ for 1 h. Data in (B–D) represent means ± SEM of three independent experiments. **P < 0.01; *P* < 0.001; *P* < 0.0001; ns, not significant; calculated by two-way ANOVA test with Tukey’s multiple-comparison posttest.

While the LVS provides an excellent model for studying *F. tularensis* pathogenesis, findings obtained with attenuated strains do not always recapitulate in human virulent strains (53 - 55). Therefore, we assayed whether the human virulent Schu S4 strain similarly dampens MAPK activation and TLR2 signaling via TolC during infection of BMMs, as observed for the LVS. To examine MAPK responses, BMMs were infected with WT or ∆*tolC* Schu S4, or left uninfected, and phosphorylated, and total levels of p38, SAPK/JNK, and ERK1/2 were determined at 30 and 60 min p.i. Infection with the Schu S4 ∆*tolC* mutant triggered increased levels of phospho-p38 at both time points when compared to macrophages infected WT Schu S4 or uninfected controls (Fig. 4; Fig. S2). This agrees with the results obtained with the LVS. Increased SAPK/JNK phosphorylation was also observed for BMMs infected with the Schu S4 ∆*tolC* mutant (Fig. 4; Fig. S2). In contrast to the LVS, increased ERK1/2 phosphorylation was additionally observed at 30 min p.i. for BMMs infected with the Schu S4 ∆*tolC* mutant (Fig. 4; Fig. S2). These results show that MAPK signaling is broadly activated early during infection of macrophages by the human virulent *F. tularensis* Schu S4 strain, and the bacteria dampen this host response in a TolC-dependent manner.

We next tested whether TLR2 signaling is required for the downstream cell death response of macrophages to Schu S4 infection, as found for the LVS. WT or TLR2^−/−^ BMMs were infected with WT or ∆*tolC* Schu S4, and cell death was measured by LDH release at 24 h p.i. Infection of WT BMMs with the Schu S4 ∆*tolC* mutant led to an approximately sixfold increase in cell death compared to infection with WT bacteria (Fig. 4B). However, the increased cytotoxicity of the ∆*tolC* mutant was no longer observed in the TLR2^−/−^ BMMs (Fig. 4B). In agreement with this, intracellular replication of the Schu S4 ∆*tolC* mutant was rescued in the TLR2^−/−^ vs WT BMMs (Fig. 4C). Thus, the *F. tularensis* LVS and Schu S4 strain both delay macrophage cell death by interfering with TLR2-dependent signaling to maintain the intracellular niche and maximize bacterial replication.

In addition to its ability to delay programmed cell death pathways during infection, the Schu S4 strain dampens pro-inflammatory cytokine responses of BMMs in a TolC-dependent manner (23). We, therefore, examined the role of TLR2 signaling in the downstream cytokine response elicited by the SchuS4 ∆*tolC* mutant during BMMs infection. WT or TLR2^−/−^ BMMs were infected with WT or ∆*tolC* Schu S4, or left uninfected, and release of the pro-inflammatory cytokine IL-6 into the culture medium was determined by ELISA at 24 h p.i. As previously observed (23), the Schu S4 ∆*tolC* mutant elicited elevated release of IL-6 (Fig. 4D) compared to infection with WT Schu S4 or uninfected controls. However, no increase in IL-6 levels was observed for infection of the TLR2^−/−^ BMMs (Fig. 4D). Thus, TLR2 is integral to the macrophage pro-inflammatory response to infection by the human virulent *F. tularensis* Schu S4 and the bacteria disrupt TLR2 signaling via TolC to suppress cytokine responses of the host.

### The LVS ∆*tolC* mutant regains full virulence in MYD88^−/−^ mice

**Fig 5:**
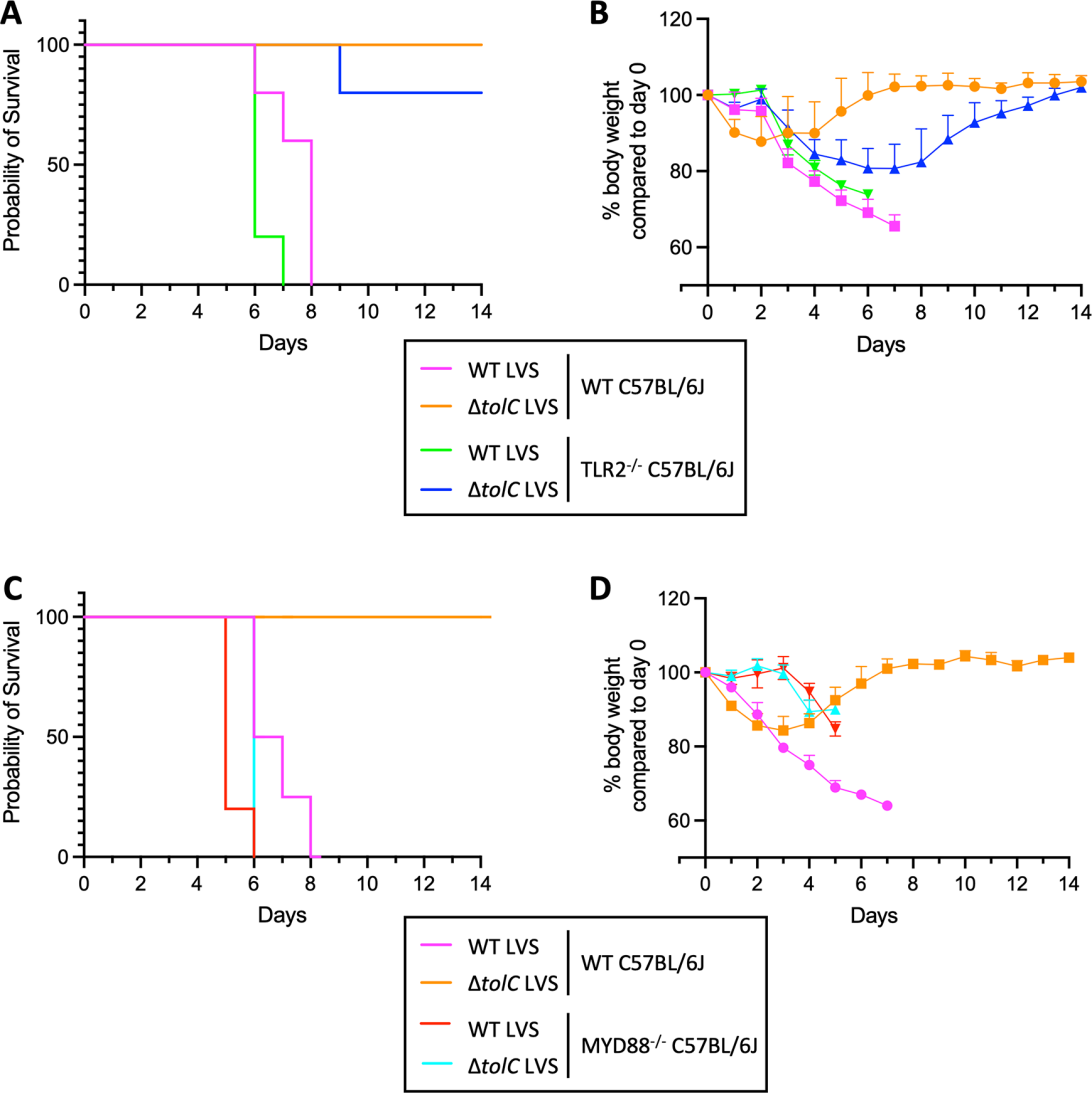
Roles of TLR2 and MYD88 in host protection during LVS intranasal infection. WT and TLR2^−/−^ (**A and B**) or MYD88^−/−^ (**C and D**) mice were infected intranasally with 5 × 10^5^ CFU of the WT or Δt*olC* LVS. The mice were monitored for survival (**A and C**) and body weight (**B and D**) for 14 d. For the WT mice in survival (**C**) and body weight (**D**), *n* = 3; for all others, *n* = 5. In (**A**), *P* = 0.0034 for comparison of WT mice infected with WT vs Δt*olC* LVS; *P* = 0.029 for comparison of WT vs TLR2^−/−^ mice infected with the WT LVS; and *P* = 0.31 for comparison of WT vs TLR2^−/−^ mice infected with the Δ*tolC* LVS. In (**C**), *P* = 0.0024 for comparison of WT mice infected with the WT vs Δ*tolC* LVS; *P* = 0.0187 for comparison of WT vs MYD88^−/−^ mice infected with the WT LVS; *P* = 0.0001 for comparison of WT vs MYD88^−/−^ mice infected with Δ*tolC* LVS; and *P* = 0.014 for comparison of MYD88^−/−^ mice infected with WT vs Δ*tolC* LVS. *P* values were calculated by the log-rank test.

Given our findings that *F. tularensis* targets TLR2- and MYD88-dependent signaling during macrophage infection, we next sought to determine the impact of this bacteria-host interplay in the mouse pneumonic tularemia model. WT and TLR2^−/−^ C57BL/6J mice were challenged by the intranasal route with 5 × 10^5^ CFU of the WT or Δ*tolC* LVS, and survival and weight loss were monitored for 14 d p.i. As expected (21, 41), WT mice infected with the WT LVS had a mean survival time of 7 d, whereas all WT mice infected with the Δ*tolC* mutant survived for the duration of the experiment (Fig. 5A and C). In comparison to the WT mice, TLR2^−/−^ mice infected with the WT LVS exhibited a faster time to death, with a mean survival time of 6 d (Fig. 5A). In TLR2^−/−^ mice infected with the Δ*tolC* LVS, one mouse succumbed to infection on day 9 p.i., while all other littermates survived (Fig. 5A). Thus, the ∆*tolC* mutant remains attenuated *in vivo* in the absence of TLR2.

Weight loss serves as an indicator of disease severity in mouse infection models (56). Consistent with the ability of *F. tularensis* to suppress host responses during the first 2–3 d p.i (17), WT mice infected with the WT LVS did not begin to exhibit weight loss until day 3 p.i., which was then followed by a steep decline in weight prior to succumbing to infection (Fig. 5B and D). In contrast, WT mice infected with the ∆*tolC* mutant exhibited weight loss starting on day 1 p.i. (Fig. 5B and D). WT mice infected with the Δ*tolC* mutant recovered from this initial decline and started to regain weight between days 4–5 p.i. Similar to WT mice, TLR2^−/−^ mice infected with the WT LVS did not begin to lose weight until day 3 p.i. (Fig. 5B). Of note, the TLR2^−/−^ mice infected with the LVS ∆*tolC* mutant no longer exhibited early weight loss on days 1–2 p.i. Instead, the TLR2^−/−^ mice exhibited an initial delay in weight loss, followed by a steep decline on days 3–4, mirroring the pattern observed for mice infected with the WT bacteria (Fig. 5B). The ∆*tolC*-infected TLR2^−/−^ mice rebounded starting on day 5 p.i., with the mice eventually regaining full body weight. Collectively, the survival and weight loss results indicate a role for TLR2 in the early response to *F. tularensis* infection; however, even in the absence of TLR2, the host is able to control infection with the LVS ∆*tolC* mutant.

We next compared infection of WT and MYD88^−/−^ mice with the WT or ∆*tolC* LVS. The infection phenotype of the MYD88^−/−^ mice was distinct from both the WT and TLR2^−/−^ mice. MYD88^−/−^ mice infected with the WT LVS exhibited a faster time to death, with a mean survival time of 5 d (Fig. 5C). Notably, the virulence of the LVS ∆*tolC* mutant was fully restored in the MYD88^−/−^ mice, with all mice succumbing to infection with kinetics similar to mice infected with the WT bacteria (Fig. 5C). The weight loss patterns for the MYD88^−/−^ mice were also distinct compared to the WT and TLR2^−/−^ mice. MYD88^−/−^ mice infected with both the WT and Δ*tolC* LVS exhibited an extended delay in weight loss, with the mice maintaining full weight until day 4 p.i. Additionally, by the time the WT and Δ*tolC* infected MYD88^−/−^ mice started to lose weight, both groups quickly succumbed to infection. Together, these data demonstrate a critical role for MYD88-dependent pathways in host protection against *F. tularensis* infection and suggest that, in the absence of MYD88, TolC-mediated suppression of host inflammatory responses is no longer required for bacterial virulence.

## DISCUSSION

*F. tularensis* delays both programmed cell death and pro-inflammatory cytokine responses during intracellular infection to ensure its replication and survival. TLR2- and MYD88-dependent signaling pathways are central regulators of the pro-inflammatory response to *F. tularensis* (26 - 30 - 38 - 40). However, mechanisms by which *F. tularensis* modulates programmed cell death responses during infection are still not understood despite being fundamental to virulence. We previously established that the outer membrane channel protein TolC is important for the ability of both the LVS and human virulent Schu S4 strain to delay apoptosis and pro-inflammatory responses during macrophage infection (21, 23, 41, 43). In this study, we took advantage of the Δ*tolC* mutant phenotype to uncover host-signaling pathways that respond to *F. tularensis* and are disrupted by the bacteria during infection. Our results reveal that *F. tularensis* interferes with TLR2-MYD88-p38 signaling in a TolC-dependent manner to block macrophage apoptotic responses early during infection. Further, we show that this strategy is conserved between the attenuated LVS and human virulent Schu S4 strain. Comparison of WT and ∆*tolC* LVS in the mouse pneumonic tularemia model revealed distinct roles for TLR2 and MYD88 in early responses to infection and host survival. MYD88-deficient mice were unable to maintain protection against infection with the LVS Δ*tolC* mutant, identifying MYD88 as critical for host defense and a key target for *F. tularensis* subversion.

*F. tularensis* induces early MAPK activation in both murine and human macrophages that is dampened by ~2 h p.i. although conflicting reports exist on the extent of MAPK activation during *Francisella* infection (13, 16, 33 - 37 - 57 - 59). Our results indicate that *F. tularensis* dampens MAPK activation during infection of macrophages as soon as 30 min p.i., through a TolC-dependent mechanism. MAPK activation then decreases by 1–2 h p.i. for both the WT and Δ*tolC F. tularensis*. The ability of *Francisella* to dampen MAPK activation has been attributed to phagosomal escape to the cytosol, where the bacteria are protected from TLR2 signaling (16, 60). The LVS was also previously shown to downregulate MAPK activation beginning at ~2 h p.i., by activating PI3 kinase during infection and increasing expression of MAPK phosphatase-1 (MKP-1) (61). Thus, *F. tularensis* likely employs several strategies to dampen MAPK activation during infection. Our results suggest that TolC-dependent activity counteracts early TLR2-MYD88 signaling, while the bacteria are still within the phagosome. Subsequent downmodulation of MAPK activity then occurs independent of TolC due to rapid exit from the phagosome together with increased MKP-1 expression.

The activation of p38 and SAPK/JNK is commonly associated with the induction of apoptosis (32, 44). Many intracellular pathogens have evolved to block p38 signaling to prevent apoptosis, and the use of p38 inhibitors during infection of monocyte-derived cells by the LVS was shown to reduce host cell death (36, 45). In line with these observations, we found that inhibiting p38 activity early during infection reduced cytotoxicity of the LVS Δ*tolC* mutant in macrophages. In contrast, treatment with the SAPK/JNK inhibitor did not alter the Δ*tolC* mutant phenotype. The increased SAPK/JNK phosphorylation we observed at 30 min p.i. suggests that SAPK/JNK is likely to perform other functions in the early host response to *F. tularensis*, such as the expression of cytokines, and the individual role of each MAPK warrants further investigation.

During *F. tularensis* infection of macrophages, both TLR2- and NADPH oxidase-dependent redox signaling lead to p38 activation (13, 33, 34, 61). Our data demonstrate that *F. tularensis* suppresses TLR2- and MYD88-dependent activation of p38 early during infection in a TolC-dependent manner. Moreover, our results show that evasion of TLR2-mediated signaling allows the bacteria to delay host apoptotic responses and thereby maximize replication in the protected intracellular niche. In contrast, we did not observe a role for the NADPH oxidase in MAPK activation or host cell death for either the WT or Δ*tolC* LVS. This is consistent with previous observations that *Francisella* inhibits the oxidative burst immediately upon infection via the activity of alkaline phosphatases and antioxidants, and that secretion of the major alkaline phosphatase AcpA occurs independently of TolC (13, 14, 62).

Like the LVS, we found that the human virulent Schu S4 strain suppresses activation of p38 and SAPK/JNK in a TolC-dependent manner. However, different from the LVS, we also observed increased ERK1/2 phosphorylation with the Schu S4 Δ*tolC* mutant. Therefore, the human virulent Schu S4 strain may more broadly activate MAPK signaling in host cells compared to the attenuated LVS. As found with the LVS, TLR2 signaling was required for the increased cytotoxicity and decreased intracellular replication observed during infection with the Schu S4 ∆*tolC* mutant. Expression of the pro-inflammatory cytokine IL-6 is also known to be induced downstream of TLR2 and p38 (26, 45, 63). In agreement with this, we found that TLR2 signaling was required for increased release of IL-6 in response to infection with the Schu S4 ∆*tolC* mutant. Together, our results identify a key role for the TLR2-p38 axis in macrophage recognition of both attenuated and human virulent strains of *F. tularensis*. Future studies will be needed to define a possible role for ERK1/2 during Schu S4 infection of macrophages and to determine if *F. tularensis* similarly activates MAPKs in human macrophages.

Despite similar requirements for TLR2 and MYD88 in controlling intracellular replication of *Francisella* in macrophages, we found that MYD88 plays a more significant role in protection than TLR2 in the mouse pneumonic tularemia model. While the TLR2^−/−^ mice exhibited a faster time to death upon infection with the WT LVS, the TLR2^−/−^ mice were still able to control and survive infection with the ∆*tolC* mutant. In contrast, not only did the MYD88^−/−^ mice succumb to infection faster with the WT LVS, but also the virulence of the ∆*tolC* mutant was fully restored. Our data agree with previous reports that MYD88 serves a broader role than TLR2 in mediating host protection against *Francisella* (26, 38 - 64 - 64). MYD88 is required for the secretion of IFN-γ in response to *F. tularensis* infection, and IFN-γ is capable of restricting the growth of virulent *F. tularensis* both *in vitro* and *in vivo* (65 - 67). MYD88-dependent IFN-γ production is independent of TLR2 and, instead, is driven by IL-18 signaling via the IL-1R (39, 40). Thus, *F. tularensis* may antagonize MYD88 to block both TLR and IL-1R signaling during infection. In the absence of MYD88, both signaling networks are disrupted, and TolC-mediated suppression of host inflammatory responses is no longer required for bacterial virulence.

Our results support a central role for TolC in the ability of *F. tularensis* to subvert TLR2-MYD88-MAPK signaling during infection. However, the mechanism by which TolC acts on these host pathways remains to be determined. We previously demonstrated that the *F. tularensis* Δ*tolC* mutant has no observable growth defect or compromised membrane integrity and that the mutant replicates intracellularly without activating caspase-1 (21, 23, 41, 43). This suggests that bacterial lysis or other structural defect is not responsible for the increased host cell death and pro-inflammatory responses triggered by infection with the ∆*tolC* mutant. Our finding that the ∆*tolC* mutant regains full virulence in mice lacking MYD88 further argues against a general role for TolC in bacterial integrity or other function. One possibility is that TolC (which is not a lipoprotein itself) may act, either directly or indirectly, to limit exposure of lipoproteins or other molecules that are recognized by TLR2 during infection. A second, non-exclusive, possibility is that TolC may disrupt host cell signaling through the secretion of effector proteins as part of a type I secretion system. Further work is needed to identify potential effectors secreted via TolC or if TolC might function through a different mechanism.

In summary, we present evidence that *F. tularensis* delays apoptosis during the infection of macrophages by limiting TLR2-MYD88-p38 signaling at the early stages of infection. This TolC-dependent activity allows *F. tularensis* to preserve its intracellular niche and prolong time for replication in the protected host cell environment. Early recognition of *F. tularensis* by TLR2 and MYD88 contributes to host protection during both macrophage and *in vivo* infection, with MYD88 playing a more critical role *in vivo. F. tularensis* subversion of TLR2-MYD88-p38 signaling is likely central to its ability to remain immunologically silent during the early stages of infection and key to its extreme virulence. Further understanding of the mechanism by which *F. tularensis* disrupts host cell signaling via TolC will enhance our understanding of the pathogenesis of tularemia and provide targets for therapeutic intervention.

## MATERIALS AND METHODS

### Bacteria and growth conditions

The *F. tularensis* LVS and SCHU S4 strain (BEI Research Resources Repository, Manassas, VA, USA) were grown on chocolate II agar plates (BD Biosciences, Franklin Lakes, NJ, USA) or in modified Mueller-Hinton broth (MHB; Mueller-Hinton broth BD Biosciences containing 1% glucose, 0.025% ferric pyrophosphate, and 0.05% l-cysteine hydrochloride). All growth and manipulations of the Schu S4 strain were performed under biosafety level 3 (BSL3) containment conditions.

### Mice

WT (C57BL/6J), TLR2^−/−^ (B6.129-*Tlr2^tm1Kir^*/J), MYD88^−/−^ (B6.129P2(SJL)-*Myd88^tm1.1Defr^*/J), and p47^phox−/−^ (B6N.129S2-*Ncf1^tm1Shl^*/J) mice were purchased from The Jackson Laboratory. Female mice were 6–8 weeks old and were used for isolation of BMMs and mouse infections.

All protocols involving animals were approved by the Institutional Animal Care and Use Committee of Stony Brook University.

### Mouse infections

Inoculums for the mouse infections were prepared by growing bacteria for 60 h on plates. Bacteria were then scraped, washed with phosphate-buffered saline (PBS), and resuspended in MHB supplemented with 10% sucrose (MHB-Sucrose). Inoculums were then serially diluted in MHB-sucrose and frozen at −80°C until use. Intranasal mouse infections were performed by administering 5.0 × 10^5^ CFU in a 10-µL inoculum into each narris (20 µL total). Actual infectious doses were determined by retrospective CFU counts. All mice were then monitored for 14 d.

### Preparation of murine macrophages

BMMs were isolated from WT, TLR2^−/−^, MYD88^−/−^, or p47^phox−/−^ C57Bl/6J mice as described (43). Bone marrow-derived cells were cultured for 6 d in bone marrow differentiation medium (BMDM; Dulbecco modified Eagle medium DMEM with GlutaMax Gibco supplemented with 30% L929 cell supernatant, 20% fetal bovine serum FBS; HyClone, and 1 mM sodium pyruvate). For immunoblotting experiments, BMMs were seeded at 1.0 × 10^6^ cells/well in 3 mL of BMDM. For cytotoxicity, replication, and ELISA assays, BMMs were seeded at 1.5 × 10^5^ cells/well in 1 mL of BMDM. BMMs were allowed to adhere to plates overnight and then washed with PBS prior to infection with the desired *F. tularensis* strains resuspended in bone marrow infection medium (BMIM; DMEM with GlutaMax Gibco supplemented with 15% L929 cell supernatant, 10% FBS, and 1 mM sodium pyruvate).

### BMM infections

For all BMM infections using the LVS, cells were seeded as described above and infected with the WT or Δ*tolC* LVS at an MOI of 50. Plates were then centrifuged at 100 × *g* for 5 min to synchronize infection. For all Schu S4 infections, BMMs were infected at an MOI of 500. Plates were not centrifuged following the addition of bacteria to prevent the generation of aerosols, necessitating the higher MOI. For intracellular replication, cytotoxicity, ELISA, and caspase-3 measurements, BMMs were infected for 2 h, washed with PBS, and incubated for an additional 1 h with 10 µg/mL of gentamicin to remove extracellular bacteria. After gentamicin treatment, cells were washed with PBS and then incubated with fresh BMIM (lacking gentamicin) until the desired time points. To measure MAPK activation, BMMs were infected for the indicated times without the addition of gentamicin. For experiments using MAPK inhibitors, BMMs were pretreated for 30 min prior to infection with 10 µM of the p38 inhibitor SB203580, 15 µM of the SAPK/JNK inhibitor SP62001, or DMSO as a vehicle control. Inhibitor concentrations were chosen based on the published literature (36, 46 - 50). We also confirmed that the addition of either inhibitor at these concentrations does not interfere with bacterial replication in broth culture. The inhibitors were removed prior to gentamicin treatment.

### Immunoblotting

To measure MAPK phosphorylation, BMMs were cultured and infected as described above. As a positive control, cells were treated with 25 ng/µL of Pam_3_CSK_4_ (Sigma, St. Louis, MO, USA). At the indicated time points, cells were washed once with cold PBS, scraped, and collected. Cells were then pelleted and lysed in radioimmunoprecipitation assay buffer containing 1 × PhosSTOP (Roche, Indianapolis, IN, USA) and 1 × cOmplete Protease Inhibitor Cocktail plus EDTA (Roche). Protein concentrations were determined by BCA assay and 5 µg of protein for each sample was separated by SDS-PAGE and transferred to nitrocellulose at 100 V for 1 h. Membranes were blocked in 5% bovine album serum in TBST (Tris-buffered saline TBS with 0.1% Tween 20) for 1.5 h at room temperature. Membranes were then incubated overnight at 4°C with primary antibodies recognizing phosphorylated p38 (Cell Signaling #4511, 1:2,000 Dilution), p38 (Cell Signaling #9212, 1:3,000 dilution), phosphorylated SAPK/JNK (Cell Signaling #9255 1:2,000 dilution), SAPK/JNK (Cell Signaling #9252, 1:3,000 dilution), phosphorylated ERK1/2 (Cell Signaling #4370, 1:2,000 dilution), ERK1/2 (Cell Signaling #4695, 1:3,000 dilution), or β-actin (Sigma; A3854, 1:50,000 dilution). Membranes were then washed with TBST and incubated with a horse radish peroxidase-conjugated anti-rabbit IgG (Cell Signaling #7074, 1:10,000 dilution) for 2 h at room temperature. To measure apoptosis, BMMs were cultured and infected as described above. Caspase-3 cleavage was detected by incubating the membranes overnight at 4°C using a primary anti-caspase-3 antibody that detects both the full-length and 17-kDa cleavage fragment (Cell Signaling, # 9661. 1:1,000 dilution). Membranes were then processed as described for the MAPK blots. All immunoblots were imaged by enhanced chemiluminescence (GE Life Sciences), and densitometric analysis was performed using ImageJ.

### Cytotoxicity assays

At 24 h p.i., supernatants from infected BMMs were collected and analyzed for LDH release using the CytoTox 96 nonradioactive cytotoxicity assay (Promega) following the manufacturer’s protocol. Supernatant fractions from uninfected BMM controls were used to determine background LDH release, and maximum LDH release was determined from BMM that were lysed via a single −80°C/37°C freeze-thaw cycle. Percent LDH release was calculated by subtracting the background LDH release from all samples, dividing the resulting sample values by the value for the maximum LDH release, and multiplying by 100.

### Intracellular replication assays

At 24 h p.i., BMMs were washed twice with PBS and then lysed in DMEM with 0.1% deoxycholate for 10 min at room temperature. Serial dilutions were then performed in PBS with the cell lysates, plated, and incubated for 3 d prior to enumeration of CFUs.

### Cytokine analysis

At 24 h p.i., culture supernatants from BMMs infected with the Schu S4 strains were collected following approved BSL3 guidelines. Levels of IL-6 were determined using a mouse IL-6 ELISA kit (Biolegend) according to the manufacturer’s protocol. Values were recorded as picograms per milliliter.

### Statistical analysis

For the LDH release, intracellular replication, and MAPK experiments, one-tailed *P* values were calculated by two-way ANOVA test with Tukey’s multiple-comparison posttest. The mouse survival studies were analyzed by the log-rank test. Statistical calculations were performed using GraphPad Prism.

## SUPPLEMENTAL MATERIAL

Supplemental Figures mbio.01136-23-s0001.pdf Figures S1 and S2.

ASM does not own the copyrights to Supplemental Material that may be linked to, or accessed through, an article. The authors have granted ASM a non-exclusive, world-wide license to publish the Supplemental Material files. Please contact the corresponding author directly for reuse.

## References

[1] Ellis J , Oyston PCF , Green M , Titball RW . 2002. Tularemia. Clin Microbiol Rev 15:631–646. doi:10.1128/CMR.15.4.631-646.2002 12364373 PMC126859

[2] Hall JD , Woolard MD , Gunn BM , Craven RR , Taft-Benz S , Frelinger JA , Kawula TH . 2008. Infected-host-cell repertoire and cellular response in the lung following inhalation of Francisella tularensis Schu S4, LVS, or U112 . Infect Immun 76:5843–5852. doi:10.1128/IAI.01176-08 18852251 PMC2583552

[3] Clemens DL , Lee BY , Horwitz MA . 2004. Virulent and avirulent strains of Francisella tularensis prevent acidification and maturation of their phagosomes and escape into the cytoplasm in human macrophages. Infect Immun 72:3204–3217. doi:10.1128/IAI.72.6.3204-3217.2004 15155622 PMC415696

[4] Roberts LM , Tuladhar S , Steele SP , Riebe KJ , Chen CJ , Cumming RI , Seay S , Frothingham R , Sempowski GD , Kawula TH , Frelinger JA . 2014. Identification of early interactions between Francisella and the host. Infect Immun 82:2504–2510. doi:10.1128/IAI.01654-13 24686053 PMC4019147

[5] Lai XH , Golovliov I , Sjöstedt A . 2001. Francisella tularensis induces cytopathogenicity and apoptosis in murine macrophages via a mechanism that requires intracellular bacterial multiplication. Infect Immun 69:4691–4694. doi:10.1128/IAI.69.7.4691-4694.2001 11402018 PMC98551

[6] Chong A , Wehrly TD , Nair V , Fischer ER , Barker JR , Klose KE , Celli J . 2008. The early phagosomal stage of Francisella tularensis determines optimal phagosomal escape and Francisella pathogenicity island protein expression . Infect Immun 76:5488–5499. doi:10.1128/IAI.00682-08 18852245 PMC2583578

[7] Sjöstedt A . 2006. Intracellular survival mechanisms of Francisella tularensis, a stealth pathogen. Microbes Infect 8:561–567. doi:10.1016/j.micinf.2005.08.001 16239121

[8] Bosio CM . 2011. The subversion of the immune system by Francisella tularensis. Front Microbiol 2:9. doi:10.3389/fmicb.2011.00009 21687406 PMC3109352

[9] Zarrella TM , Singh A , Bitsaktsis C , Rahman T , Sahay B , Feustel PJ , Gosselin EJ , Sellati TJ , Hazlett KRO . 2011. Host-adaptation of Francisella tularensis alters the bacterium’s surface-carbohydrates to hinder effectors of innate and adaptive immunity. PLoS One 6:e22335. doi:10.1371/journal.pone.0022335 21799828 PMC3142145

[10] Andersson H , Hartmanová B , KuoLee R , Rydén P , Conlan W , Chen W , Sjöstedt A . 2006. Transcriptional profiling of host responses in mouse lungs following aerosol infection with type A Francisella tularensis. J Med Microbiol 55:263–271. doi:10.1099/jmm.0.46313-0 16476789

[11] Mares CA , Ojeda SS , Morris EG , Li Q , Teale JM . 2008. Initial delay in the immune response to Francisella Tularensis is followed by Hypercytokinemia characteristic of severe sepsis and correlating with upregulation and release of damage-associated molecular patterns. Infect Immun 76:3001–3010. doi:10.1128/IAI.00215-08 18411294 PMC2446713

[12] McCrumb FR . 1961. Aerosol infection of man with Pasteurella tularensis. Bacteriol Rev 25:262–267. doi:10.1128/br.25.3.262-267.1961 16350172 PMC441102

[13] Rabadi SM , Sanchez BC , Varanat M , Ma Z , Catlett SV , Melendez JA , Malik M , Bakshi CS . 2016. Antioxidant defenses of Francisella tularensis modulate macrophage function and production of proinflammatory Cytokines. J Biol Chem 291:5009–5021. doi:10.1074/jbc.M115.681478 26644475 PMC4777838

[14] Mohapatra NP , Soni S , Rajaram MV , Dang PM , Reilly TJ , El-Benna J , Clay CD , Schlesinger LS , Gunn JS . 2010. Francisella acid phosphatases inactivate the NADPH oxidase in human phagocytes. J Immunol 184:5141–5150. doi:10.4049/jimmunol.0903413 20348422 PMC2952287

[15] Bauler TJ , Chase JC , Wehrly TD , Bosio CM . 2014. Virulent Francisella tularensis destabilize host mRNA to rapidly suppress inflammation. J Innate Immun 6:793–805. doi:10.1159/000363243 24902499 PMC4201887

[16] Telepnev M , Golovliov I , Sjöstedt A . 2005. Francisella tularensis LVS initially activates but subsequently down-regulates intracellular signaling and cytokine secretion in mouse monocytic and human peripheral blood mononuclear cells. Microb Pathog 38:239–247. doi:10.1016/j.micpath.2005.02.003 15925273

[17] Bosio CM , Bielefeldt-Ohmann H , Belisle JT . 2007. Active suppression of the pulmonary immune response by Francisella tularensis Schu4. J Immunol 178:4538–4547. doi:10.4049/jimmunol.178.7.4538 17372012

[18] Santic M , Pavokovic G , Jones S , Asare R , Kwaik YA . 2010. Regulation of apoptosis and anti-apoptosis signalling by Francisella tularensis. Microbes Infect 12:126–134. doi:10.1016/j.micinf.2009.11.003 19925880 PMC2819573

[19] Jessop F , Schwarz B , Heitmann E , Buntyn R , Wehrly T , Bosio CM . 2018. Temporal manipulation of mitochondrial function by virulent Francisella tularensis to limit inflammation and control cell death. Infect Immun 86:e00044-18. doi:10.1128/IAI.00044-18 29760217 PMC6056872

[20] Kinkead LC , Whitmore LC , McCracken JM , Fletcher JR , Ketelsen BB , Kaufman JW , Jones BD , Weiss DS , Barker JH , Allen L-AH . 2018. Bacterial lipoproteins and other factors released by Francisella tularensis modulate human neutrophil LifeSpan. Cell Microbiol 20:e12795. doi:10.1111/cmi.12795 29063667 PMC5764820

[21] Doyle CR , Pan J-A , Mena P , Zong W-X , Thanassi DG . 2014. Tolc-dependent modulation of host cell death by the Francisella Tularensis live vaccine strain. Infect Immun 82:2068–2078. doi:10.1128/IAI.00044-14 24614652 PMC3993420

[22] Wickstrum JR , Bokhari SM , Fischer JL , Pinson DM , Yeh H-W , Horvat RT , Parmely MJ . 2009. Francisella tularensis induces extensive caspase-3 activation and apoptotic cell death in the tissues of infected mice . Infect Immun 77:4827–4836. doi:10.1128/IAI.00246-09 19703976 PMC2772556

[23] Kopping EJ , Doyle CR , Sampath V , Thanassi DG . 2019. Contributions of Tolc Orthologs to Francisella Tularensis Schu S4 multidrug resistance, modulation of host cell responses, and virulence. Infect Immun 87:e00823-18. doi:10.1128/IAI.00823-18 30670554 PMC6434128

[24] Lai X-H , Sjöstedt A . 2003. Delineation of the molecular mechanisms of Francisella Tularensis-induced apoptosis in murine Macrophages. Infect Immun 71:4642–4646. doi:10.1128/IAI.71.8.4642-4646.2003 12874344 PMC165996

[25] Hajjar AM , Harvey MD , Shaffer SA , Goodlett DR , Sjöstedt A , Edebro H , Forsman M , Byström M , Pelletier M , Wilson CB , Miller SI , Skerrett SJ , Ernst RK . 2006. Lack of in vitro and in vivo recognition of Francisella tularensis subspecies lipopolysaccharide by toll-like receptors. Infect Immun 74:6730–6738. doi:10.1128/IAI.00934-06 16982824 PMC1698081

[26] Roberts LM , Ledvina HE , Sempowski GD , Frelinger JA . 2014. Tlr2 signaling is required for the innate, but not adaptive response to LVS clpB. Front Immunol 5:426. doi:10.3389/fimmu.2014.00426 25250027 PMC4155801

[27] Cole LE , Shirey KA , Barry E , Santiago A , Rallabhandi P , Elkins KL , Puche AC , Michalek SM , Vogel SN . 2007. Toll-like receptor 2-mediated signaling requirements for Francisella tularensis live vaccine strain infection of murine macrophages. Infect Immun 75:4127–4137. doi:10.1128/IAI.01868-06 17517865 PMC1951974

[28] Cole LE , Santiago A , Barry E , Kang TJ , Shirey KA , Roberts ZJ , Elkins KL , Cross AS , Vogel SN . 2008. Macrophage proinflammatory response to Francisella tularensis live vaccine strain requires coordination of multiple signaling pathways. J Immunol 180:6885–6891. doi:10.4049/jimmunol.180.10.6885 18453609 PMC2637793

[29] Malik M , Bakshi CS , Sahay B , Shah A , Lotz SA , Sellati TJ . 2006. Toll-like receptor 2 is required for control of pulmonary infection with Francisella tularensis. Infect Immun 74:3657–3662. doi:10.1128/IAI.02030-05 16714598 PMC1479238

[30] Abplanalp AL , Morris IR , Parida BK , Teale JM , Berton MT . 2009. TLR-dependent control of Francisella tularensis infection and host inflammatory responses. PLoS One 4:e7920. doi:10.1371/journal.pone.0007920 19936231 PMC2775407

[31] Balka KR , De Nardo D . 2019. Understanding early TLR signaling through the Myddosome. J Leukoc Biol 105:339–351. doi:10.1002/JLB.MR0318-096R 30256449

[32] Yue J , López JM . 2020. Understanding MAPK signaling pathways in apoptosis. Int J Mol Sci 21:2346. doi:10.3390/ijms21072346 32231094 PMC7177758

[33] Dai S , Rajaram MV , Curry HM , Leander R , Schlesinger LS . 2013. Fine tuning inflammation at the front door: macrophage complement receptor 3-mediates phagocytosis and immune suppression for Francisella tularensis. PLoS Pathog 9:e1003114. doi:10.1371/journal.ppat.1003114 23359218 PMC3554622

[34] Edwards MW , Aultman JA , Harber G , Bhatt JM , Sztul E , Xu Q , Zhang P , Michalek SM , Katz J . 2013. Role of mTOR downstream effector signaling molecules in Francisella tularensis Internalization by murine macrophages. PLoS One 8:e83226. doi:10.1371/journal.pone.0083226 24312679 PMC3849438

[35] Saint RJ , D’Elia RV , Bryant C , Clark GC , Atkins HS . 2016. Mitogen-activated protein kinases (Mapks) are modulated during Francisella tularensis infection, but inhibition of extracellular-signal-regulated kinases (Erks) is of limited therapeutic benefit. Eur J Clin Microbiol Infect Dis 35:2015–2024. doi:10.1007/s10096-016-2754-1 27714591 PMC5138274

[36] Brummett AM , Navratil AR , Bryan JD , Woolard MD , R. Blanke S . 2014. Janus kinase 3 activity is necessary for phosphorylation of cytosolic phospholipase A2 and prostaglandin E2 synthesis by macrophages infected with Francisella tularensis live vaccine strain. Infect Immun 82:970–982. doi:10.1128/IAI.01461-13 24343645 PMC3957991

[37] Hrstka R , Stulík J , Vojtesek B . 2005. The role of MAPK signal pathways during Francisella tularensis LVS infection-induced apoptosis in murine Macrophages. Microbes Infect 7:619–625. doi:10.1016/j.micinf.2004.12.020 15820149

[38] Collazo CM , Sher A , Meierovics AI , Elkins KL . 2006. Myeloid differentiation Factor-88 (Myd88) is essential for control of primary in vivo Francisella Tularensis LVS infection, but not for control of intra-macrophage bacterial replication. Microbes Infect 8:779–790. doi:10.1016/j.micinf.2005.09.014 16513388

[39] Skyberg JA , Lacey CA . 2017. Hematopoietic Myd88 and IL-18 are essential for IFN-gamma-dependent restriction of type A Francisella Tularensis infection. J Leukoc Biol 102:1441–1450. doi:10.1189/jlb.4A0517-179R 28951422 PMC5669634

[40] Pietras EM , Miller LS , Johnson CT , O’Connell RM , Dempsey PW , Cheng G . 2011. A Myd88-dependent Ifngammar-Ccr2 signaling circuit is required for mobilization of monocytes and host defense against systemic bacterial challenge. Cell Res 21:1068–1079. doi:10.1038/cr.2011.59 21467996 PMC3193491

[41] Gil H , Platz GJ , Forestal CA , Monfett M , Bakshi CS , Sellati TJ , Furie MB , Benach JL , Thanassi DG . 2006. Deletion of Tolc Orthologs in Francisella Tularensis identifies roles in multidrug resistance and virulence. Proc Natl Acad Sci U S A 103:12897–12902. doi:10.1073/pnas.0602582103 16908853 PMC1568944

[42] Costa TRD , Felisberto-Rodrigues C , Meir A , Prevost MS , Redzej A , Trokter M , Waksman G . 2015. Secretion systems in gram-negative bacteria: structural and mechanistic insights. Nat Rev Microbiol 13:343–359. doi:10.1038/nrmicro3456 25978706

[43] Platz GJ , Bublitz DC , Mena P , Benach JL , Furie MB , Thanassi DG . 2010. A tolC mutant of Francisella Tularensis is Hypercytotoxic compared to the wild type and elicits increased proinflammatory responses from host cells. Infect Immun 78:1022–1031. doi:10.1128/IAI.00992-09 20028804 PMC2825903

[44] Gräb J , Rybniker J . 2019. The expanding role of P38 mitogen-activated protein kinase in programmed host cell death. Microbiol Insights 12:1178636119864594. doi:10.1177/1178636119864594 31384128 PMC6657118

[45] Arthur JSC , Ley SC . 2013. Mitogen-activated protein Kinases in innate immunity. Nat Rev Immunol 13:679–692. doi:10.1038/nri3495 23954936

[46] Bennett BL , Sasaki DT , Murray BW , O’Leary EC , Sakata ST , Xu W , Leisten JC , Motiwala A , Pierce S , Satoh Y , Bhagwat SS , Manning AM , Anderson DW . 2001. Sp600125, an Anthrapyrazolone inhibitor of Jun N-terminal kinase. Proc Natl Acad Sci U S A 98:13681–13686. doi:10.1073/pnas.251194298 11717429 PMC61101

[47] Aguiló N , Uranga S , Marinova D , Martín C , Pardo J . 2014. Bim is a crucial regulator of apoptosis induced by Mycobacterium tuberculosis. Cell Death Dis 5:e1343. doi:10.1038/cddis.2014.313 25032866 PMC4123102

[48] Alemán M , Schierloh P , de la Barrera SS , Musella RM , Saab MA , Baldini M , Abbate E , Sasiain MC . 2004. Mycobacterium tuberculosis triggers apoptosis in peripheral neutrophils involving toll-like receptor 2 and P38 mitogen protein kinase in tuberculosis patients. Infect Immun 72:5150–5158. doi:10.1128/IAI.72.9.5150-5158.2004 15322009 PMC517458

[49] Deng L , Chen M , Tanaka M , Ku Y , Itoh T , Shoji I , Hotta H . 2015. HCV upregulates bim through the ROS/JNK signalling pathway, leading to BAX-mediated apoptosis. J Gen Virol 96:2670–2683. doi:10.1099/jgv.0.000221 26296767

[50] Wang JH , Zhou YJ , He P , Chen BY . 2007. Roles of mitogen-activated protein kinase pathways during Escherichia coli-induced apoptosis in U937 cells. Apoptosis 12:375–385. doi:10.1007/s10495-006-0623-6 17191113

[51] Jackson SH , Gallin JI , Holland SM . 1995. The P47Phox mouse knock-out model of chronic granulomatous disease. J Exp Med 182:751–758. doi:10.1084/jem.182.3.751 7650482 PMC2192153

[52] Yang CS , Shin DM , Lee HM , Son JW , Lee SJ , Akira S , Gougerot-Pocidalo MA , El-Benna J , Ichijo H , Jo EK . 2008. Ask1-P38 MAPK-P47Phox activation is essential for inflammatory responses during tuberculosis via Tlr2-ROS signalling. Cell Microbiol 10:741–754. doi:10.1111/j.1462-5822.2007.01081.x 18028450

[53] Lindgren H , Shen H , Zingmark C , Golovliov I , Conlan W , Sjöstedt A . 2007. Resistance of Francisella tularensis strains against reactive nitrogen and oxygen species with special reference to the role of KatG. Infect Immun 75:1303–1309. doi:10.1128/IAI.01717-06 17210667 PMC1828546

[54] Kadzhaev K , Zingmark C , Golovliov I , Bolanowski M , Shen H , Conlan W , Sjöstedt A . 2009. Identification of genes contributing to the virulence of Francisella Tularensis SCHU S4 in a mouse intradermal infection model. PLoS One 4:e5463. doi:10.1371/journal.pone.0005463 19424499 PMC2675058

[55] Qin A , Scott DW , Thompson JA , Mann BJ . 2009. Identification of an essential Francisella tularensis subsp. tularensis virulence factor. Infect Immun 77:152–161. doi:10.1128/IAI.01113-08 18981253 PMC2612291

[56] Olfert ED , Godson DL . 2000. Humane endpoints for infectious disease animal models. ILAR J 41:99–104. doi:10.1093/ilar.41.2.99 11406703

[57] Nakayasu ES , Tempel R , Cambronne XA , Petyuk VA , Jones MB , Gritsenko MA , Monroe ME , Yang F , Smith RD , Adkins JN , Heffron F . 2013. Comparative phosphoproteomics reveals components of host cell invasion and post-transcriptional regulation during Francisella infection. Mol Cell Proteomics 12:3297–3309. doi:10.1074/mcp.M113.029850 23970565 PMC3820940

[58] Huang MT-H , Mortensen BL , Taxman DJ , Craven RR , Taft-Benz S , Kijek TM , Fuller JR , Davis BK , Allen IC , Brickey WJ , Gris D , Wen H , Kawula TH , Ting JP-Y . 2010. Deletion of ripA Alleviates suppression of the Inflammasome and MAPK by Francisella Tularensis. J Immunol 185:5476–5485. doi:10.4049/jimmunol.1002154 20921527 PMC4671501

[59] Parsa KVL , Butchar JP , Rajaram MVS , Cremer TJ , Tridandapani S . 2008. The tyrosine kinase Syk promotes phagocytosis of Francisella through the activation of Erk. Molecular Immunology 45:3012–3021. doi:10.1016/j.molimm.2008.01.011 18295889 PMC2408723

[60] Cole LE , Laird MHW , Seekatz A , Santiago A , Jiang Z , Barry E , Shirey KA , Fitzgerald KA , Vogel SN . 2010. Phagosomal retention of Francisella Tularensis results in TIRAP/mal-independent Tlr2 signaling. J Leukoc Biol 87:275–281. doi:10.1189/jlb.0909619 19889726 PMC2812562

[61] Medina EA , Morris IR , Berton MT . 2010. Phosphatidylinositol 3-kinase activation attenuates the Tlr2-mediated macrophage proinflammatory cytokine response to Francisella tularensis live vaccine strain. J Immunol 185:7562–7572. doi:10.4049/jimmunol.0903790 21098227

[62] Hoang KV , Chen CG , Koopman J , Moshiri J , Adcox HE , Gunn JS . 2016. Identification of genes required for secretion of the Francisella oxidative burst-inhibiting acid phosphatase AcpA. Front Microbiol 7:605. doi:10.3389/fmicb.2016.00605 27199935 PMC4848305

[63] Fitzgerald KA , Kagan JC . 2020. Toll-like receptors and the control of immunity. Cell 180:1044–1066. doi:10.1016/j.cell.2020.02.041 32164908 PMC9358771

[64] De Pascalis R , Rossi AP , Mittereder L , Takeda K , Akue A , Kurtz SL , Elkins KL . 2020. Production of IFN-gamma by splenic dendritic cells during innate immune responses against Francisella tularensis LVS depends on Myd88. PLoS One 15:e0237034. doi:10.1371/journal.pone.0237034 32745117 PMC7398525

[65] Edwards JA , Rockx-Brouwer D , Nair V , Celli J . 2010. Restricted cytosolic growth of Francisella tularensis subsp. tularensis by IFN-gamma activation of macrophages. Microbiology (Reading) 156:327–339. doi:10.1099/mic.0.031716-0 19926654 PMC2890092

[66] Jessop F , Buntyn R , Schwarz B , Wehrly T , Scott D , Bosio CM , Roy CR . 2020. Interferon gamma reprograms host mitochondrial metabolism through inhibition of complex II to control intracellular bacterial replication. Infect Immun 88:e00744-19. doi:10.1128/IAI.00744-19 31740527 PMC6977132

[67] Anthony LS , Morrissey PJ , Nano FE . 1992. Growth inhibition of Francisella tularensis live vaccine strain by IFN-gamma-activated macrophages is mediated by reactive nitrogen intermediates derived from L-arginine metabolism. J Immunol 148:1829–1834. 1541823

