# Supplementary material for: Francisella tularensis disrupts TLR2-MYD88-p38 signaling early during infection to delay apoptosis of macrophages and promote virulence in the host
Source: mBio. 2023 Jul 5;14(4):e01136-23. doi: 10.1128/mbio.01136-23 (PMC10470500; doi:10.1128/mbio.01136-23)
Supplement: Supplemental Figures — Figures S1 and S2. [file mbio.01136-23-s0001.pdf]

Francisella tularensis disrupts TLR2-MYD88-p38 signaling early during infection to delay apoptosis of macrophages and promote virulence in the host

P. Todd Benziger, Erik J. Kopping, Patrick A. McLaughlin, David G. Thanassi*

SUPPLEMENTAL MATERIAL

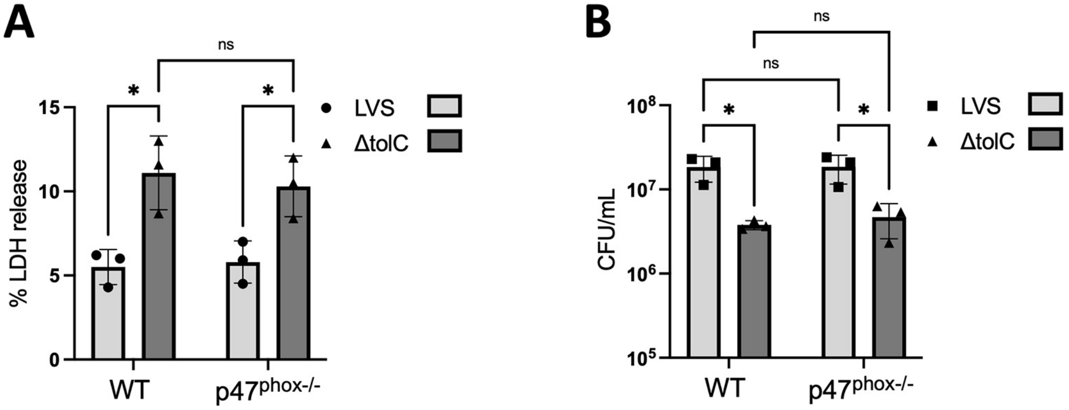

Figure S1. Loss of NADPH oxidase activity does not affect cytotoxicity and intracellular replication during LVS infection. BMM isolated from WT or p47$^{phox-/-}$ mice were infected at an MOI of 50 with the WT or Δ*tolC* LVS, or left uninfected. At 24 h p.i., supernatant fractions were collected and assayed for levels of LDH release (A) or cell lysates were plated for CFU (B). Data represent means ± SEM of three independent experiments. *P* < 0.05; **, *P* < 0.01; ns, not significant; calculated by two-way ANOVA with Tukey's multiple-comparison posttest.

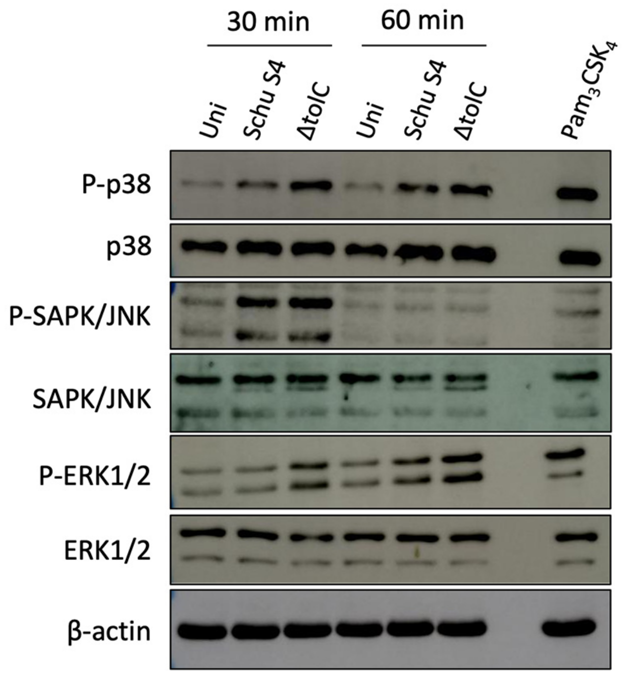

Figure S2. MAPK activation during Schu S4 infection. BMM were infected at an MOI of 500 with WT or Δ*tolC* SCHU S4, or left uninfected. At the indicated time points, macrophage lysates were collected and assayed for phosphorylation of p38, SAPK/JNK, and ERK1/2 by SDS-PAGE and immunoblotting. As a positive control, BMM were treated with 25 ng/μl of Pam_3CSK_4 for 1 h. A representative blot is shown; an additional blot is shown in Fig. 4A.
